# An Ontology for Digital Medicine Outcomes: Development of the Digital Medicine Outcomes Value Set (DOVeS)

**DOI:** 10.2196/67589

**Published:** 2025-02-06

**Authors:** Benjamin Rosner, Matthew Horridge, Guillen Austria, Tiffany Lee, Andrew Auerbach

**Affiliations:** 1 Division of Clinical Informatics and Digital Transformation University of California San Francisco San Francisco, CA United States; 2 Division of Hospital Medicine University of California San Francisco San Francisco, CA United States; 3 Stanford Center for Biomedical Informatics Research Stanford University Stanford, CA United States; 4 School of Public Health University of California Berkeley Berkeley, CA United States

**Keywords:** digital health, digital medicine, digital therapeutics, ontology, medical informatics, value set, ontology, development, digital health tool, DHT, health systems, digital medicine outcomes value set, prototype, users

## Abstract

**Background:**

Over the last 10-15 years, US health care and the practice of medicine itself have been transformed by a proliferation of digital medicine and digital therapeutic products (collectively, digital health tools [DHTs]). While a number of DHT classifications have been proposed to help organize these tools for discovery, retrieval, and comparison by health care organizations seeking to potentially implement them, none have specifically addressed that organizations considering their implementation approach the DHT discovery process with one or more specific outcomes in mind. An outcomes-based DHT ontology could therefore be valuable not only for health systems seeking to evaluate tools that influence certain outcomes, but also for regulators and vendors seeking to ascertain potential substantial equivalence to predicate devices.

**Objective:**

This study aimed to develop, with inputs from industry, health care providers, payers, regulatory bodies, and patients through the Accelerated Digital Clinical Ecosystem (ADviCE) consortium, an ontology specific to DHT outcomes, the Digital medicine Outcomes Value Set (DOVeS), and to make this ontology publicly available and free to use.

**Methods:**

From a starting point of a 4-generation–deep hierarchical taxonomy developed by ADviCE, we developed DOVeS using the Web Ontology Language through the open-source ontology editor Protégé, and data from 185 vendors who had submitted structured product information to ADviCE. We used a custom, decentralized, collaborative ontology engineering methodology, and were guided by Open Biological and Biomedical Ontologies (OBO) Foundry principles. We incorporated the Mondo Disease Ontology (MONDO) and the Ontology of Adverse Events. After development, DOVeS was field-tested between December 2022 and May 2023 with 40 additional independent vendors previously unfamiliar with ADviCE or DOVeS. As a proof of concept, we subsequently developed a prototype DHT Application Finder leveraging DOVeS to enable a user to query for DHT products based on specific outcomes of interest.

**Results:**

In its current state, DOVeS contains 42,320 and 9481 native axioms and distinct classes, respectively. These numbers are enhanced when taking into account the axioms and classes contributed by MONDO and the Ontology of Adverse Events.

**Conclusions:**

DOVeS is publicly available on BioPortal and GitHub, and has a Creative Commons license CC-BY-SA that is intended to encourage stakeholders to modify, adapt, build upon, and distribute it. While no ontology is complete, DOVeS will benefit from a strong and engaged user base to help it grow and evolve in a way that best serves DHT stakeholders and the patients they serve.

## Introduction

Over the last 10-15 years, the US health care industry and the practice of medicine itself have been transformed by a proliferation of digital health software applications. It has been estimated, for example, that more than 350,000 health and wellness apps are available in mainstream app stores [1], and that more than 300 million people have used them in one form or another [2]. Along with this breadth of applications comes a variety of terminology describing them. The colloquially used umbrella term “digital health,” for example, encapsulates many concepts including wellness apps, consumer grade mobile health apps, personal health tracking devices, remote patient monitoring applications, telemedicine platforms, and software as a medical device [3].

Driven in part by regulatory agencies and industry advocacy groups in the United States and abroad, efforts have been undertaken to more clearly delineate the boundaries between these types of applications. For example, “digital health” has been defined as technologies, platforms, and systems that engage consumers for lifestyle, wellness, and health-related purposes; capture, store, or transmit health data; and/or support life science and clinical operations [4]. As such, digital health products typically do not require clinical evidence, and do not meet the regulatory definition of a medical device. Digital *medicine* products, on the other hand, include evidence-based software and/or hardware products that measure and/or intervene in the service of human health. These products require clinical evidence and may (or may not) be classified as medical devices. At the most regulated end of this spectrum, digital *therapeutics* are health software intended to treat or alleviate a disease, disorder, condition, or injury by generating and delivering a medical intervention that has a demonstrable positive therapeutic impact on patient health and produce real-world outcomes [5]. Digital therapeutics typically do fall under regulatory oversight, with prescription digital therapeutics as those that are prescribed by a licensed health care professional.

While these definitions organize digital health tools (DHTs), a term we use here for convenience to encapsulate digital medicine and digital therapeutic products into regulatory and potentially clinical versus nonclinical categories, they do not necessarily enable the side-by-side outcomes-based comparison of similar products for the purpose of understanding relative efficacy or safety, nor do they facilitate establishing, for regulators and for vendors filing 510(k) applications, whether certain products may be “substantially equivalent” to predicate devices [6,7]. This need to evaluate evidence of health technologies and compare them with predicate devices partially overlaps with the purpose of Health Technology Assessment (HTA) frameworks, formal, systematic processes for synthesizing and evaluating existing evidence for health technologies, often for the use of policy and decision makers [8]. Users of HTA frameworks (whether federal bodies abroad or individual stakeholders in the United States where no centralized federal HTA body exists [9]) might also benefit from a means to readily identify similar or related devices, particularly in a domain as complex and rapidly evolving as digital medicine. Furthermore, most DHT end users (eg, clinicians and patients) or decision makers (eg, health system leaders making subscription or purchasing decisions, digital pharmacy and therapeutic committees making formulary decisions, or payers making coverage decisions), have one or more *outcomes* in mind that they are hoping the digital application can be used to influence.

While a few taxonomies do exist that categorize DHTs on a limited set of functional categories and characteristics [10,11], these are narrow in scope, and lack the interrelationships of an ontology. Establishing DHT organizational frameworks as ontologies provides a solution to heterogeneity problems like this, thereby defining the concepts and relationships that make interoperability possible [12]. It is common, for example, for ontologies to have “off the shelf” tooling; logic-based, precisely defined semantics; modeling languages to describe complex relationships; machine processing capability allowing for the computation of relationships; rigorous relationship structures, and in many cases the ability to be incorporated into other ontologies. In these respects, a system to organize DHTs according to the outcomes they intend to influence, and a common set of outcome metrics, would be valuable.

The purpose of this work is to describe the development of an *outcomes-based* ontology, the Digital medicine Outcomes Value Set (DOVeS). This ontology came into being as part of the Accelerated Digital Clinical Ecosystem (ADviCE) consortium, a group of health systems, vendors, payers, policymakers, and patient advocacy groups whose mission is to enable and scale the safe and effective adoption of DHTs in clinical practice [13].

## Methods

### Phase 0: Defining Scope and Purpose

DOVeS emanated from needs of the University of California San Francisco-Stanford-FDA (Food and Drug Administration) Center of Excellence in Regulatory Science and Innovation-funded ADviCE consortium to identify real-world performance metrics with which the safety and efficacy of DHTs could be characterized, whether for health care system leaders seeking to comparatively assess and implement DHTs, or for regulators seeking to classify them into similar outcomes-based groupings [14]. The purpose of DOVeS was to establish common sets of DHT real-world performance measure (RWPM) outcomes to facilitate more straightforward comparison between DHT products. The scope of DOVeS was to capture a body of outcomes with the potential to be influenced by DHTs, preferably but not exclusively by products capable of integrating into the electronic health record (EHR). DOVeS was intended to satisfy questions posed to vendors in a Digital Health Common Application (DHCA, refer to “Phase 1: PreDOVeS Development [Background Context and Knowledge Capture]” section), and to address competency questions for use cases such as: what DHT applications influence (specific outcome) pertaining to disease X, what DHT applications satisfy health care administrative outcome Y, what DHT applications potentially mitigate adverse event Z?

### Phase 1: PreDOVeS Development (Background Context and Knowledge Capture)

DOVeS emanated from ADviCE, about which background context will be helpful. Briefly, in order to inform a RWPM framework, leaders from 5 health systems participating in ADviCE formulated a strategy to learn from the DHT marketplace and collaboratively developed a Digital Health Common Application. The purpose of the DHCA was to collect from vendors a standardized set of intake and discovery information about their products and the populations and disease areas these products serve. Information collected through the DHCA across many vendors could then empower health system decision makers with a variety of common metrics in 1 place as their health systems, or any other, embark upon initial DHT vendor discovery for implementation decisions. Fields captured in the DHCA included, for example: whether the DHT integrates with the EHR (and if so, which ones); mechanisms of integration (eg, application programming interfaces, flat files, and data warehouses); current or planned regulatory status of the DHT product; stage of company development (eg, conceptual, proof of concept, beta, or commercial deployment); security, privacy, and data sharing policies; disease indications; intended patient populations; device integration; safety and efficacy evidence; accessibility, equity, feature types; and the outcomes that the product seeks to improve.

Between April 2019 and June 2020, a total of 185 vendors voluntarily completed and submitted DHCA data to ADviCE. Based on these data, on the FDA’s Developing Software Precertification Program: A Working Model [15], and on consensus-building work carried out by the ADviCE consortium, a RWPM taxonomical hierarchy was initially developed, with 3 top-level DHT classes: “Healthcare outcomes,” “Non-clinical product performance outcomes,” and “User experience outcomes.” Beneath these top-level classes were 3 levels of descendant outcome classes. The taxonomy resided in a spreadsheet, but without the functionalities of an ontology.

### Phase 2: Development of DOVeS Ontology

#### Ontology Engineering Methodology

A number of Ontology Engineering Methodologies (OEMs) exist to guide and structure ontology development from a state of informal knowledge to formal representation [16]. Many OEMs have been organized into different classes, including, for example, collaborative, noncollaborative, and custom OEMs [17]. Our approach, a custom, decentralized, collaborative OEM, emanated from the asynchronous nature of information acquisition and stakeholder input that spanned the course of the ADviCE project (a portion of which preceded DOVeS in which DHT vendors supplied data that would later inform the top-level classes of the ontology) and the DOVeS project itself (in which domain experts and engineers collaborated synchronously and iteratively with a consortium of stakeholders).

Specifically, our custom approach resembles aspects of the Human-Centered Ontology Engineering Methodology framework, emphasizing agile, collaborative, and community-driven ontology development, involving both “knowledge engineers” and “knowledge workers” (eg, DHT vendors) empowering the latter to participate iteratively in the ontology lifecycle leveraging their experience and their work setting-specific domain expertise [18]. This is consistent with recent trends in ontology engineering, and avoids rigidly defined workflows and phases, instead focusing on iterative development and leveraging tools like GitHub and Protégé to facilitate decentralized collaboration. Our synchronous, active collaboration with communities of practice included the ADviCE consortium consisting of partners from academic medical centers; community health systems; policy makers; patient advocacy groups; industry members including DHT vendors, pharma, and payers; and domain experts, an ontology engineer, and an additional round of DHT vendors during field testing (refer to Phase 3 below). Asynchronous collaboration included input from the 185 DHT vendors who completed and submitted DHCAs before formal DOVeS development began. Supported by GitHub for versioning and issue tracking, the open-source ontology editor Protégé for editing [19], and BioPortal for publishing [20], DOVES reflects principles of liveness, evolution, and reusability.

#### Content Addition

Beginning with the 3 top-level classes and the collection of descendant outcomes described above, we used the web ontology language (OWL) through the open-source ontology editor Protégé to create an ontology, and to populate subclasses, definitions, and axioms related to DHTs. Based on data supplied by the 185 vendors, input from members of the ADviCE consortium, 1 investigator’s first-hand knowledge of the digital health space [BR], and 2 investigators’ involvement in the University of California San Francisco’s Digital Diagnostics and Therapeutics Committee, a digital Pharmacy and Therapeutics committee [21] [BR and AA], we added more structure including subclasses, definitions, relationships, and refining values.

#### Technical Considerations

The DOVeS ontology was developed specifically using the OWL2EL fragment. This fragment is commonly used in many biomedical ontologies due to its balance between expressivity and computational efficiency [22]. Throughout the development process, we aimed to follow the Open Biological and Biomedical Ontologies (OBO) Foundry principles [23]. While these principles do not themselves constitute a methodology, they represent well-established community standards that when used in conjunction with a methodology, help ensure that the processes for creating and maintaining DOVeS would be robust, scalable, interoperable, and aligned with widely accepted ontology development best practices [23]. Since DOVeS is specific to digital medicine and therapeutic applications, and not a general-purpose biomedical domain ontology, there are no plans to submit it to the OBO Foundry Library.

Of the OBO Foundry’s 15 principles, the following 6 directly influenced our development environment and setup (Table 1), while other principles such as P8: documentation, P10: commitment to collaboration, and several others were used for management and maintenance, and are accommodated through our use of GitHub and BioPortal.

**Table 1:** Open Biological and Biomedical Ontologies Foundry principles directly influencing Digital medicine Outcomes Value Set development environment and setup.

OBO^a^ Foundry principle	Description
P1: Open	DOVeS^b^ ontology sources and releases are publicly available on GitHub under the open and permissive CC-BY-SA Creative Commons license.
P2: Common Format	We represented DOVeS using OWL^c^. OWL supports serialization into several accepted concrete syntaxes, including the OBO syntax, allowing interoperability with various ontology tools. This choice aligns with the OBO principle of adhering to a common format across ontologies.
P3: URI^d^/Identifier Space	Uniform naming conventions were applied to the DOVeS ontology Resource Identifier and all associated terms. Each term is assigned a universally unique alphanumeric identifier, prefixed by “https://w3id.org/doves/.” This approach follows the OBO Foundry recommendation to use a meaningless alpha-numeric identifier for ontology terms, with human-readable labels encoded separately, as noted above.
P4: Versioning	GitHub served as the primary platform for ontology development, providing out-of-the-box versioning and release functionality. Our development approach closely followed the established ontology engineering practices popularized by the Gene Ontology Consortium [24,25]. Released versions of DOVeS are made publicly available on BioPortal to support accessibility and dissemination [20].
P6: Textual Definitions	Terms within DOVeS are accompanied by textual definitions, which follow the Aristotelian genus-differentia descriptive format. This format defines a term by specifying its broader category (genus) and its distinguishing characteristics (differentia). These definitions are provided using the IAO:0000115 (definition) annotation property.
P12: Naming Conventions	We applied consistent naming conventions across all DOVeS terms. Specifically, terms are named in lowercase unless they are proper nouns, and each term has exactly 1 primary label specified by an “rdfs:label” annotation. In addition, synonyms and alternative labels are provided using “skos:altLabel” annotations.

^a^OBO: Open Biological and Biomedical Ontologies.

^b^DOVeS: Digital medicine Outcomes Value Set.

^c^OWL: Web Ontology Language.

^d^URI: Unique Resource Identifier.

#### Secondary Ontology Integrations

Because many DHTs are meant to influence diseases or disease trajectories, we incorporated the Mondo Disease Ontology (MONDO), an OWL ontology unifying multiple disease ontologies and terminologies. MONDO is a publicly available ontology with an active maintenance userbase that harmonizes multiple disease resources [26]. It consists of 54,000 classes of which the human disease branch has 22,500 classes. We extracted the human disease branch (MONDO:0700096) using the *minimum information to reference an external ontology term* (MIREOT) method [27] provided by the ROBOT command line tool [28]. We chose MONDO over the *ICD-10* (*International Classification of Diseases, Tenth Revision*), for example, because MONDO is a richly axiomatized OWL ontology whereas *ICD-10* is a structured taxonomy that is optimized for health care reporting and billing. Using MONDO, therefore, allowed us to leverage the built-in semantics of OWL and an OWL reasoner to infer relationships between disease terms and for runtime querying. Furthermore, terms in MONDO are, to a greater degree than *ICD-10*, richly annotated with synonyms and definitions, including synonyms from *ICD-10*. This makes MONDO more useful for DOVeS at runtime where applications may need to search for DHTs by synonyms. Finally, MONDO worked well with our build tools. We used the ODK (Ontology Development Kit), which works best with OBO-style ontologies, like MONDO.

Because many DHTs influence or improve outcomes by mitigating adverse events, feedback from collaborators strongly emphasized the importance of addressing adverse events in a manner that would be both scalable and interoperable. We therefore incorporated the Ontology of Adverse Events (OAE) [29]. The OAE is an OBO Foundry ontology available in OWL, which ensures compatibility with and facilitates seamless integration into DOVeS*.* This allows DHTs to be described in terms of outcomes that correspond to mitigation of certain types of adverse events such as the number or rate of diabetic hypoglycemic events (OAE:0001057).

### Phase 3: Testing and Refinement

After initial ontology development based on input from the ADviCE consortium, internal expertise, and DHCA data of vendors in the ADviCE database, the DOVeS ontology was then externally field tested with an independent sample of DHT vendors who had not been through the DHCA intake process and were not otherwise familiar with it. Lists of potential vendors for outreach were provided by ADviCE collaborators, the Digital Therapeutics Alliance, and ORCHA (Organization for the Review and Care of Health Apps). Inclusion criteria consisted of vendors serving the US market with digital medicine or digital therapeutic products that in some way served patients, clinicians, or both, for clinical care and were not purely administrative in nature.

To carry out this field testing, we reached out by email to 206 new DHT vendors from the above lists between December 2022 and May 2023. Nonresponses were followed up with an additional 2 outreach emails of varying invitation language. A total of 40 vendors replied, expressing interest (19.4% response rate), and were subsequently invited to one-on-one semistructured interviews (Multimedia Appendix 1) by web meetings to allow them to visually inspect the ontology in a dynamic manner in Protégé, and to suggest additions and modifications. Thirty-seven of these vendors (92.5%), 17 of which were manufacturers of FDA-listed Artificial Intelligence/Machine Learning Medical Devices, accepted this invitation and participated in the web meetings. To plan for these meetings and to facilitate quickly honing in on the areas within the ontology of greatest relevance to each of the vendors during the limited time of the 1 hour web meetings, vendors were sent REDCap (Research Electronic Data Capture; Vanderbilt University) surveys in advance (Multimedia Appendix 2), asking them to describe the areas in which their products operated and the types of outcomes their products aimed to influence, including: clinical outcomes, educational outcomes (eg, patient comprehension), engagement and adherence outcomes (eg, user engagement), health economic outcomes, health care operations outcomes (eg, operational efficiency), health care utilization outcomes (eg, hospital readmission), patient reported outcomes (eg, patient reported outcome response rates), process of care outcomes (eg, adherence to HEDIS [Healthcare Effectiveness Data and Information Set] measures), cybersecurity (eg, security and privacy certifications), interoperability, EHR integration, user experience (eg, Net Promoter Score), product uptime or downtime outcomes, and any peer reviewed manuscripts describing outcomes. Vendors could respond to 1 or more of these areas, or to none of them, thereby providing investigators insight as to where within the ontology to begin the semistructured interview. Of the 40 vendors sent REDCap surveys, 26 (65%) completed them before meeting with us. Those that did not complete them, but that nevertheless participated in one-on-one web meetings (11), had opportunities to describe their outcomes and areas of focus verbally. Information gleaned from the web meetings were captured in a database for potential DOVeS modifications.

Before new ontology information was to be captured through these meetings, we preidentified a stopping point for the one-on-one vendor meetings, as the point in time at which a “steady state” had been achieved in the top-level classes and their 3 descendent generations (ie, the original hierarchy from ADviCE plus any updates from the Phase 2 development process). This steady state was defined as the absence of additional suggestions for modification to these classes in the top 4 generations from no less than 5 sequential vendors. Any single vendor suggestion to modify one of these high-level classes constituted a signal to continue to interview vendors, record their suggestions, and make modifications as indicated. The stopping point of 5 vendors in a row without modification suggestions to these top classes was achieved by the 35th one-on-one meeting, but we nevertheless completed the remaining vendor meetings that had already been scheduled. Although some vendors late in the process had no ontology modification suggestions at all (suggesting some degree of ontology maturity even at deeper generation levels), many had highly use-case specific outcomes suggestions particular to their products at generations greater than 3 descendants deep from the top class. Although no ontology can be considered “complete,” we took this trend in modification suggestions as a sign of reasonable high-level class content and structural stability.

### Phase 4: Prototype Development for Real-World Application Demonstration

To demonstrate the potential use of DOVeS for practical application, we built a prototype DHT Application Finder that allows a user to search for DHTs based on filter settings, outcomes-based search terms, conditions, specialties, procedures, types of technology, regulatory status, and EHR integration capabilities. The backend of the DHT Application Finder used OWL reasoning in particular standard subsumption tests, to compute answers to queries from the frontend. To identify specific DHTs, the DHT Application Finder referenced the ADviCE database of 185 vendors’ products. The records in the database were annotated with ontology terms from DOVeS that described the conditions treated by the application, outcomes of relevance, and other product parameters that were available in the original data collected by the DHCA. At runtime we constructed an OWL class expression that ontologically described each DHT application. When a user submits a search request, the criteria in the search request are translated to a complex OWL class expression that describes a general class of DHT applications. Then OWL reasoning is used to classify and retrieve the DHT applications that are entailed to be subclasses of the general class.

## Results

### Phases 0-3 Scope, Background, Development, Refinement, and Testing (Principal Results)

Despite the large set of classes pertaining directly to health care outcomes, “Non-clinical product performance outcomes” and “User experience outcomes” were nevertheless felt to be important not only to health systems and regulators, but to DHT vendors themselves. These classes consisted respectively of (1) technical outcomes associated with the product (cybersecurity and privacy certification outcomes), interoperability, technical certification, and system availability outcomes to name a few, and (2) a variety of outcomes associated with user satisfaction and usability.

**Figure 1:**
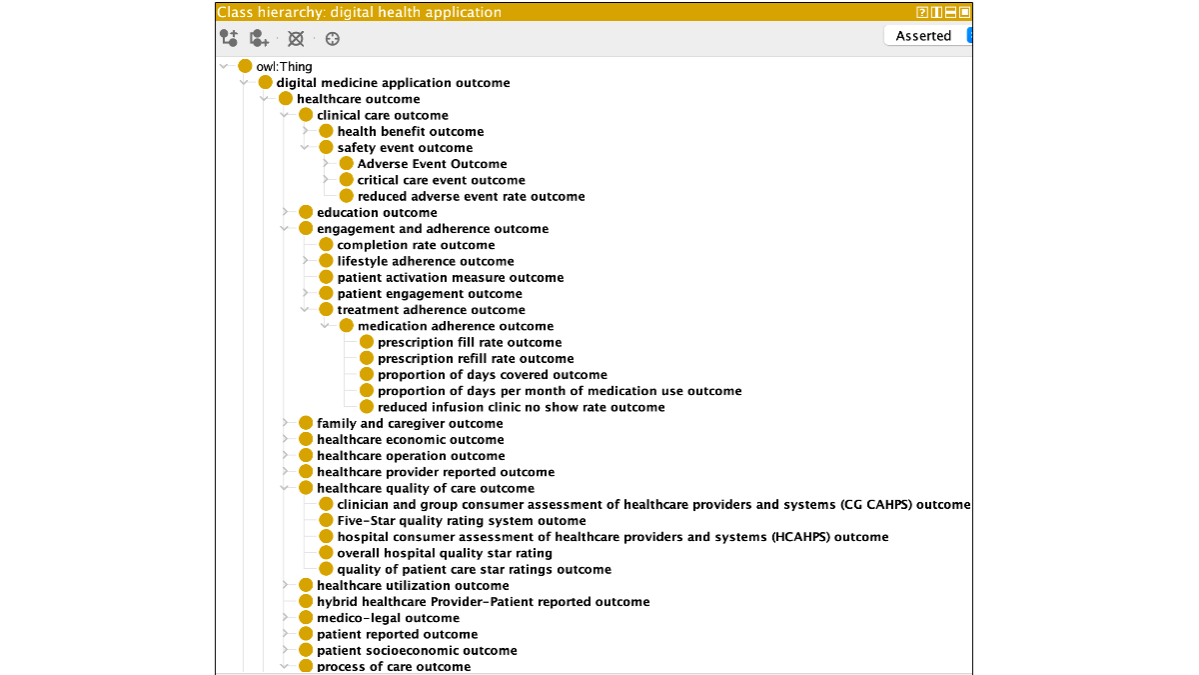
A selected hierarchical segment of Digital medicine Outcomes Value Set from the Protégé development environment.

In its current state, DOVeS contains 42,320 and 9481 native axioms and distinct classes respectively (Table 2). These numbers are enhanced when taking into account the axioms and classes contributed by MONDO and the OAE. A static view of a selected portion of the ontology hierarchy is shown in Figure 1. Dynamic views of the full ontology can be explored at BioPortal [30].

**Table 2:** Digital medicine Outcomes Value Set summary metrics.

Metric	Count native to DOVeS^a^	Count including MONDO^b^ and OAE^c^
Axioms	42,320	286,097
Number of distinct, named classes	9481	36,412
Object properties	12	32

^a^DOVeS: Digital medicine Outcomes Value Set.

^b^MONDO: Mondo Disease Ontology.

^c^OAE: Ontology of Adverse Events.

### Phase 4 Prototype Development (Principal Results)

**Figure 2:**
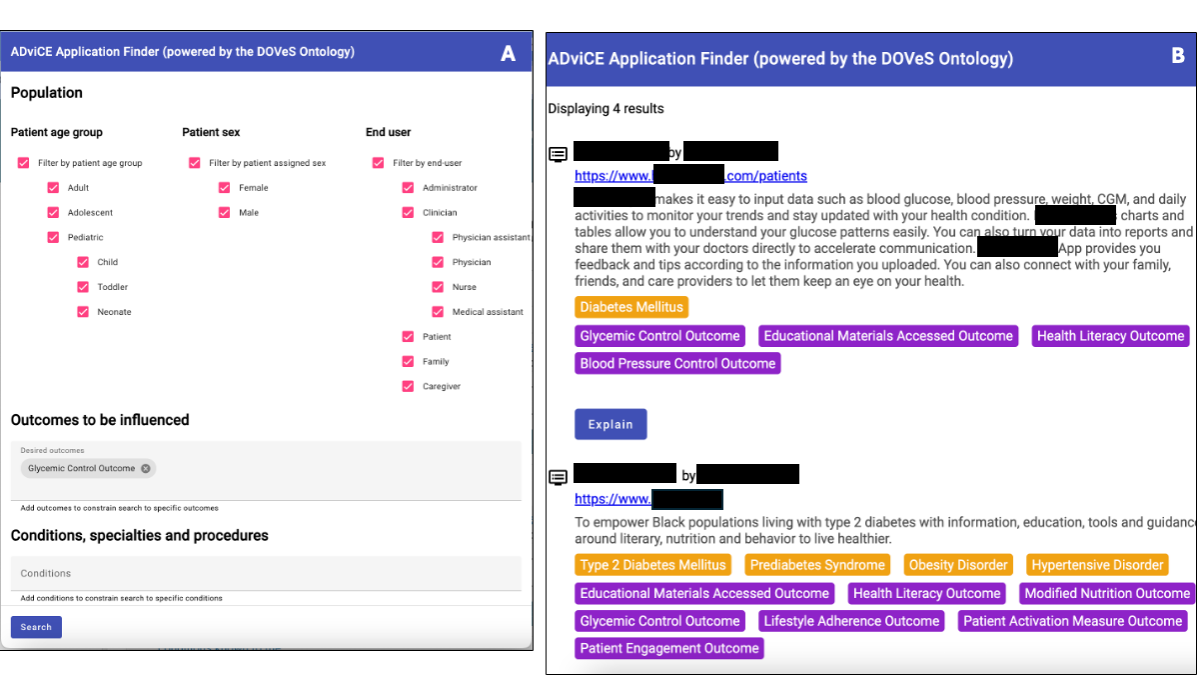
Prototype Digital Health Tool Application Finder with underlying DOVeS (Digital medicine Outcomes Value Set) ontology. (A) Search for outcome “glycemic control.” (B) A set of search results. (Company and product names redacted).

**Figure 3:**
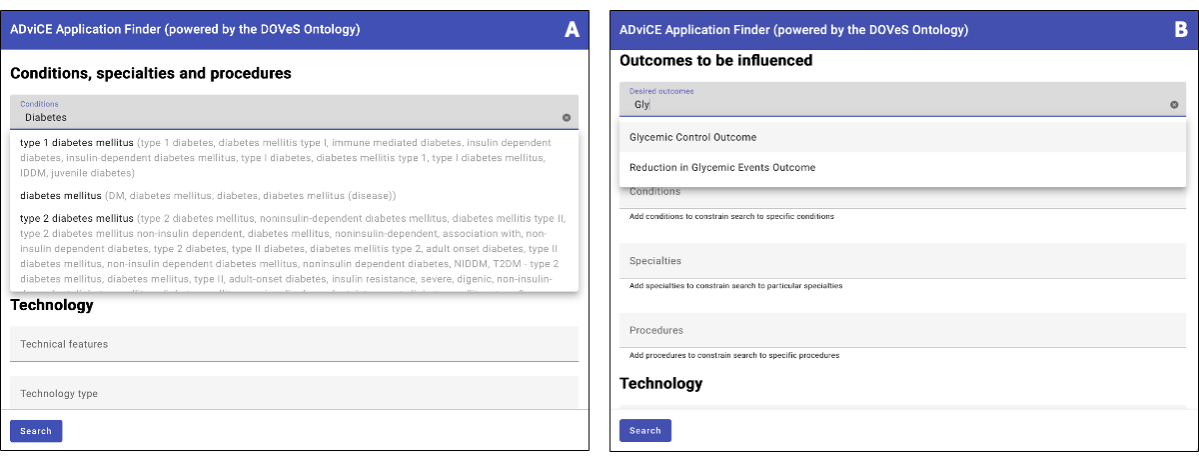
Entry fields in the Digital Health Tool Application Finder for (A) conditions, specialties, and procedures, and (B) outcomes to be influenced. The fields show type-ahead completion options for conditions known to the system, along with their synonyms that are drawn directly from Mondo Disease Ontology (MONDO).

The prototype DHT Application Finder front end and sample search results are shown in Figure 2. The DHT Application Finder’s functionality is best illustrated with an example. Because of the availability of multiple DHTs in the ADviCE database pertaining to diabetes, we describe a use case in which a health system decision maker seeks to find candidate DHTs to help patients with diabetes in their health system achieve glycemic control (ie, glycemic control is the outcome of interest). The example is most informative by illustrating an end user seeking to identify DHTs first without, and then with outcomes-based search terms. In this example, the end user initially searches for DHTs based only on “diabetes” in the Conditions field. (Figure 3A) The user selects “diabetes mellitus” from the type-ahead list that appears, and presses “Search.” The search results show a list of DHTs associated with diabetes mellitus including those for “type 2 diabetes mellitus” as well as “diabetic retinopathy.” However, neither of these provides a list of DHTs exclusively related to the outcome of glycemic control.

Therefore, the user, in addition, (or could have done so as the first step) enters the term “Glyc” into the “Outcomes to be influenced” entry field (Figure 3B), and the type-ahead offers 2 choices: “Glycemic Control” and “Reduction in Glycemic Events.” Choosing “Glycemic Control Outcome” and performing the search again, the user obtains a list now of only those DHTs specific to achieving glycemic control (eg, this time not including DHTs associated specifically with diabetic retinopathy). In the case of the ADviCE database, this outcomes-based search reduced the field of products that the user might otherwise have needed to explore further by 33%.

## Discussion

### Principal Results

In developing DOVeS, we describe an ontology framed by outcomes specific to DHTs, rather than technical or implementation features. Because our goal was to address products that would potentially be used in clinical practice that might fall under regulatory oversight, we focused on digital medicine and digital therapeutic products, and did not intend to capture outcomes associated with the much broader category of digital health.

The 3 top-level classes of DOVeS, derived from the background work done by the ADviCE consortium, consisted of “Healthcare outcomes,” “Non-clinical product performance outcomes,” and “User experience outcomes.” The top-level class “Healthcare outcomes” is central to DHTs, as most such products intend to influence something in this class. It is composed of descendent classes including, for example, clinical care outcomes (eg, health benefits and mitigation of adverse events), health care utilization outcomes, health care operational outcomes, process of care outcomes, medico-legal outcomes, education outcomes, health care economic outcomes, socioeconomic outcomes, family and caregiver outcomes, and engagement and adherence outcomes (Figure 1).

### Related Works

Efforts to organize DHTs to date have been described in part based on the specialties they serve, disease areas, user types, and certain functionalities [31], but this can limit the way in which DHTs that are platforms, serving functions across multiple specialties, or that span diseases of multiple organ systems (eg, diabetes) are organized. The World Health Organization developed the Classifications of Digital Health Interventions as a nonontological framework according to different user types (persons, health care providers, health management, and support personnel), data services, and services and application types, but not on outcomes, and not in a computable format [32].

Although some limited outcomes-based taxonomies [33] and classifications exist [34], to our knowledge, none are specific to digital medicine or digital therapeutics applications. Organizing DHTs into common categories based on the outcomes they influence then gives the marketplace an opportunity to compare different DHTs side by side for their impacts on common outcomes, and with the appropriate outcomes results, may make regulatory, purchasing, formulary, and coverage decisions more efficient and objective.

### Limitations

Several limitations to this work exist. First, by design, DOVeS was informed by DHTs excluding those in the “digital health” class (that class of products focused on wellness that does not fall under regulatory oversight). Second, while it would be methodologically satisfying after DOVeS development to return to the original 185 DHT vendor products and quantitatively describe the degree of concept mapping achieved, doing so could be misleading because the only data available to us from these vendors were high-level concepts captured in the DHCAs before DOVeS was built. Therefore, a future and important direction for the DHT Application Finder will be a guided intake module through which the vendor can submit a product for automated annotation based on the dynamic class content of the ontology at the time of submission. Another limitation is that, while the classes for the ontology were developed with academic, regulatory, community health, payer, and patient advocacy stakeholders, and included field testing with an independent set of DHT vendors, DOVeS, like most ontologies, is not complete, but will rely and depend on an active base of engaged supporters to continue to build and maintain it. In addition, outcomes and the metrics to measure those outcomes are not necessarily universal in the DHT industry. In the process of outcome and metrics discovery, we attempted to select outcomes through both internal and external consensus. However, these outcomes and metrics may need modification in the future as the ontology grows and is further informed by the broader community of stakeholders and the evolving industry.

### Conclusions

An ontology for organizing DHTs into outcomes-based groupings is an important step as the digital medicine and digital therapeutics industry matures. While the DOVeS ontology provides an open-source framework as a starting point to do this, real-world value from the ontology will be achieved through the development of robust tools similar to the prototype DHT Application Finder that will leverage the ontology for specific use cases. These may include, for example, use by regulators to streamline the process for determining substantial equivalence to predicate devices, and use by health system decision makers to identify products that influence the same or related outcomes so that the universe of potential DHT products being considered for implementation can be rapidly narrowed to only those that are most relevant. While it is not known whether the 33% reduction in products that were more specifically identified with an outcomes-based search relative to a disease-based search described in the example from the ADviCE database above will necessarily translate if the entire marketplace of DHT products were to be indexed, any reasonable reduction would nevertheless offer savings in time and effort by decision makers attempting to identify DHTs for deeper discovery for potential clinical use.

DOVeS is publicly available on BioPortal [30] and GitHub [35], and has a Creative Commons license CC-BY-SA that is intended to encourage stakeholders to modify, adapt, build upon, and distribute it. Only with an engaged userbase will the ontology continue to grow and evolve, and when applied to the marketplace help organize DHTs in a way that is beneficial to stakeholders and ultimately to the patients they serve.

## Appendix

Multimedia Appendix 1 Semi-structured interview questions for web meetings with Digital Health Tool vendors.

Multimedia Appendix 2 REDCap survey sent to vendors in advance of web meetings in order to help investigators focus on the most relevant parts of the ontology for the web meeting.

## Abbreviations

ADviCE: Accelerated Digital Clinical Ecosystem
DHCA: Digital Health Common Application
DHT: digital health tool
DOVeS: Digital medicine Outcomes Value Set
EHR: electronic health record
FDA: Food and Drug Administration
HEDIS: Healthcare Effectiveness Data and Information Set
HTA: Health Technology Assessment
ICD-10: International Classification of Diseases, Tenth Revision
MIREOT: minimum information to reference an external ontology term
MONDO: Mondo Disease Ontology
OAE: Ontology of Adverse Events
OBO: Open Biological and Biomedical Ontologies
ODK: Ontology Development Kit
OEM: Ontology Engineering Methodology
ORCHA: Organization for the Review and Care of Health Apps
OWL: Web Ontology Language
REDCap: Research Electronic Data Capture
RWPM: Real World Performance Metrics
