# Supplementary material for: An Ontology for Digital Medicine Outcomes: Development of the Digital Medicine Outcomes Value Set (DOVeS)
Source: JMIR Med Inform. 2025 Feb 6;13:e67589. doi: 10.2196/67589 (PMC11843056; doi:10.2196/67589)
Supplement: Multimedia Appendix 1 [file medinform_v13i1e67589_app1.docx]

DOVeS Semi-Structured Interview Questions of Independent Set of Vendors

1. What are the names of your company and your company’s digital health tool application(s)?
2. In 2-3 sentences, please generally describe the use and goals of your digital health tool application(s).
3. What disease areas does/do your application(s) aim to address?
4. What outcomes does/do your application(s) seek to improve?
5. [If relevant, and while displaying the visual OntoGraph of the ontology] Which of the following outcomes does/do your application(s) measure, track, and/or seek to improve? If any that describe your application(s) are missing, please suggest additions.
   1. Clinical Care
   2. User Experience
   3. Educational outcomes (patient, provider, etc.)
   4. Engagement or adherence
   5. Product Performance (e.g. uptime/downtime, cybersecurity certifications, interoperability standards adherence, issue resolution metrics, electronic health record integration(s), regulatory certifications/standards)
   6. Healthcare economics
   7. Healthcare operations
   8. Healthcare utilization
   9. Patient reported outcomes
   10. Processes of care
6. What technologies or features does/do your application(s) use (e.g. artificial intelligence, capture of patient generated health data, etc.)?
7. What peer reviewed publications, if any, are available describing outcomes with respect to your application(s)?
8. What comments do you have regarding the ontology, including suggestions for changes to content or structure?
