# Supplementary material for: An Ontology for Digital Medicine Outcomes: Development of the Digital Medicine Outcomes Value Set (DOVeS)
Source: JMIR Med Inform. 2025 Feb 6;13:e67589. doi: 10.2196/67589 (PMC11843056; doi:10.2196/67589)
Supplement: Multimedia Appendix 2 [file medinform_v13i1e67589_app2.pdf]

Vendor reported measures

Please complete the survey below.

Thank you!

1) Company name: __________________________________

2) Product or application name: __________________________________

3) Clinical care outcomes:
Does the product claim a direct and MEASURABLE impact on a patient's disease state (e.g. improving HbA1c in diabetics, mitigating surgical site infections, etc.). If so, please specify the disease(s) and HOW the measurement is measured (e.g. the equation that describes the measured impact). ____

4) Education outcomes:
Does the product claim a direct and MEASURABLE impact on educational outcomes (e.g. patient comprehension is increased by X%, or provider knowledge about a given disease is increased by Y%)? If so, please specify the name of the educational outcome (e.g. "Patient comprehension") and HOW it is measured (e.g. the equation that described the measured impact). ____

5) Engagement and adherence outcomes:
Does the product claim a direct and MEASURABLE impact on a user's engagement (e.g. patient's have an average (SD) engagement of X%, or physician users have an average (SD) engagement of Y%) If so, please specify the HOW the measurement is measured (e.g. the equation that describes the measured impact). ____

6) Healthcare economic outcomes:
Does the product claim a direct and MEASURABLE impact on a healthcare economic outcome (e.g. $ saved per patient per year, cost/quality adjusted life year, return on investment, etc.) If so, please specify the name of the economic outcome(s) (e.g. $ saved per patient per year) and HOW the measurement is measured (e.g. the equation that describes the measured impact). ____

7) Healthcare operation outcomes:
Does the product claim a direct and MEASURABLE impact on a healthcare operations outcome (e.g. improving back office efficiency, reducing phone call volumes, saving time, etc.) If so, please specify the operations outcome and HOW the measurement is measured (e.g. the equation that describes the measured impact). ____

8) Healthcare utilization outcome:
Does the product claim a direct and MEASURABLE impact on a healthcare utilization outcome (e.g. all cause 30 day hospital readmission rate, 30-day ED visit rate, etc.). If so, please specify the type of healthcare utilization outcome (be as specific as possible; for example, rather than readmission rate, and rather than 30-day readmission rate, please list all cause 30-day readmission rate) and HOW the measurement is measured (e.g. the equation that describes the measured impact). __________________________________

9) Patient-reported outcomes:
Does the product claim a direct and MEASURABLE impact on a patient reported outcome metric (PROM) For example, PROM response rate, change in a PROM metric over time (e.g. X% increase in 30-day KOOS score post-total knee arthroplasty). If so, please specify the PROM, the disease(s) and HOW the measurement is measured (e.g. the equation that describes the measured impact). __________________________________

10) Process of care outcomes:
Does the product claim a direct and MEASURABLE impact on an process of care outcome (e.g. greater adherence to HEDIS process of care measures, Y% increase in adherence to diabetic eye exam, etc.). If so, please specify the process of care that is impacted, and HOW the measurement is measured (e.g. the equation that describes the measured impact). __________________________________

11) Cybersecurity:
Please describe the cybersecurity measures in place for the product. For example, are data stored and transmitted in an encrypted manner, are data servers restricted, are data servers in the U.S. or abroad; is the privacy of user data protected properly; is user data sharing appropriate; is there an ongoing process for ensuring security, do you adhere to any security standards? __________________________________

12) Interoperability standards:
Does the product integrate with user's digital health ecosystem (e.g. consumer electronic devices/trackers, smartphone, etc.). If so, please specify.
Does the product align with larger data standards (e.g., USCDI)? __________________________________

13) EHR integration:
For example, does the product integrate with EHRs (which ones)? __________________________________

14) Issue resolution:
Does the product or the company track issue resolution metrics (e.g. expected vs actual response time; issue resolution time) If so, please describe the name of the metric tracker, and HOW the metric is measured (an equation that describes the measurement. For example, Average Monthly Issue Resolution Time = Sum (time issue resolved - time issue filed) / number of issues filed in a calendar month).

____

15) User experience:
Does the product report a standard way of measuring user experience (e.g. Net Promoter Score, star rating, etc.)? If so, which user experience metrics do you use, and how to you report them (aggregate over all time, annual calendar year, rolling annual, rolling monthly average, etc.).

____

16) Uptime/downtime:
Does the company track product uptime or downtime? If so, which one?
How is it reported (e.g. Aggregate over all time, annual calendar year, rolling annual, rolling monthly, etc.).

____

17) Manuscripts:
If the product has any scholarly manuscripts (e.g. those in the peer reviewed literature) that support any of its claims, please enter the citation (or PUBMED ID) here.

____
